# Actively expressed microbiota in mucosal biopsies of treatment-naïve ulcerative colitis patients

**DOI:** 10.1080/29933935.2025.2512763

**Published:** 2025-06-05

**Authors:** Line Strand Karlsholm, Rafi Ahmad, Hagar Taman, Christopher G. Fenton, Ruth H. Paulssen

**Affiliations:** a Clinical Bioinformatics Research Group, Department of Clinical Medicine, Faculty of Health Sciences, UiT- The Arctic University of Norway, Tromsø, Norway; b Genomics Support Centre Tromsø, UiT- The Arctic University of Norway, Department of Clinical Medicine, Faculty of Health Sciences, UiT- The Arctic University of Norway, Tromsø, Norway; c Department of Biotechnology, University of Inland Norway, Hamar, Norway

**Keywords:** Meta-transcriptomics, ulcerative colitis, gut microbiota, inflammation

## Abstract

The gut microbiota contributes to an aberrant immune dysfunction associated with inflammatory bowel disease (IBD) and is believed to contribute to the disease development of ulcerative colitis (UC). In this study, a whole-transcriptomic data set containing mucosal tissue biopsies obtained from treatment-naïve UC patients (*n* = 33) and control subjects (*n* = 15) were utilized to determine potential differences in microbial compositions located at the site of inflammation. Transcriptomic sequences that mapped to the human genome were removed from the dataset. The remaining were mapped to their respective Operational Taxonomic Units (OTUs) resulting in an overview of the microbial composition at the genus level. Results showed an overall decrease in microbial diversity in mucosal UC patient samples. However, an increase of the opportunistic pathogens *Acinetobacter* and *Comamonas* was observed in UC samples. *Comamonas* is an environmental pathogen. In addition to their role in mild but persistent infections, *Comamonas* species may play a role in active UC. The experimental approach described here provides new insights into the UC microbiota composition directly at the site of inflammation, which could contribute to a better understanding of UC pathogenesis. The obtained results might provide an opportunity to investigate host–microbe interactions in active UC.

## Introduction

The gut microbiota is a known factor in inflammatory bowel disease (IBD) pathogenesis.^1,2^ Ulcerative colitis (UC) is a chronic inflammatory condition of the colon and rectum and one of the major phenotypes of IBD.^3–5^ The microbial community present in the colon plays a role in maintaining homeostasis in the colon. An aberrant intestinal flora is thought to play a key role in triggering inflammation in UC.^1,6^ This study aimed to determine specific changes in the gut microbial profile of treatment-naïve UC patients at the site of inflammation by an alternative meta-transcriptomics approach. Usually, for characterizing the gut microbiome, metagenomics approaches like 16S rRNA gene and shotgun DNA sequencing have widely been used.^7–11^ Both methods are standard methods for the identification of microbes at the genus or species level. Meta-transcriptomics, the analysis of actively transcribed bacterial RNA in colonic samples, has been used less frequently to study the gut microbiome.^7,12^ In contrast to 16S rRNA gene and shotgun DNA sequencing, the study of actively transcribed genes in meta-transcriptomics analyses can reveal important interactions between microbes and their hosts and how expressed functions can influence disease progression and severity in UC patients.^11,13,14^ Fecal samples have often been the preferred sample type when studying the gut microbiota in UC due to the non-intrusive collection method. However, fecal samples might include microorganisms that do not directly interact with the mucosa as they contain microorganisms from the entire gastrointestinal (GI) tract.^15^

This study used mucosal biopsies and whole-transcriptomic data from patients with active UC for meta-transcriptomics analysis. It is believed that this approach provides a better characterization of the microbes that are directly involved at the site of inflammation thereby permitting the characterization of functionally active bacteria in the colon of UC patients.^16,17^

## Materials and methods

### Characteristics of the study cohort

The study cohort comprised transcriptomic data of mucosal biopsies from patients with newly diagnosed, treatment-naïve UC (*n* = 33) and control subjects (C) (*n* = 15).^18,19^ For controls, biopsies from subjects undergoing cancer screening with normal colonoscopy and normal colonic histological examination were used. All patients and control subjects underwent the same standard colonoscopy preparation routinely used at the hospital, and biopsies were taken from the sigmoid part of the colon. UC was diagnosed based upon established clinical endoscopic and histological criteria as defined by the ECCO (European Crohn’s and Colitis Organisation) guidelines.^20^ The biopsies from UC samples showed clinical Mayo scores with disease activity index (UCDAI) of 8,2 ± 2,5 standard deviation (SD). None of the patients included in this study were using antibiotics or probiotics. The participants signed an informed and written consent form. The study was approved by the Regional Ethics Committee of North Norway and the Norwegian Social Science Data Services (REK Nord 2012/1349). The samples were taken from an established biobank approved by the Norwegian Board of Health (952/2006), the Advanced Study of Inflammatory Bowel Disease (ASIB) study at the University Hospital of Northern Norway (UNN).

### RNA isolation, library preparation & next generation sequencing

Mucosal tissue samples were taken at the site of inflammation by a standardized endoscopic method with biopsy forceps 2.8 mm. All samples were taken from the sigmoid part of the colon with most inflammation and immediately stored in RNALater (Qiagen) for further downstream analysis. All samples were stored at room temperature overnight and then at −20°C until RNA isolation. RNA was isolated from homogenized tissue using the Allprep DNA/RNA Mini Kit from Qiagen (Cat no: 80204) and the QIAcube instrument (Qiagen), according to the manufacturer’s protocol. RNA quantity and purity were assessed as previously described.^19,21^ All RNA samples used for analyses had a RIN value between 8.0 and 10.0. Whole transcriptome libraries were prepared with the TruSeq Stranded Total RNA LT Sample Prep Kit from Illumina (Catalogue number RS-122–2203) and as previously described.^19^ The libraries were subsequently paired end sequenced (150 bp) with the NextSeq 550 instrument (Illumina). Library preparations and sequencing were conducted as described previously.^19^

### Data analysis

Quality control and adapter trimming was done directly by the Illumina NextSeq550’s built-in analysis software. As a result, adapter sequences and sequences with a Phred score under 30 were removed from the output fastq files. The quality checked and trimmed reads were then mapped to the human genome assembly 38 (GRCh38.p12) using bowtie2 (version 3.2.5.1) short-read mapper with the very-sensitive mode. The sequences that mapped to the human genome were excluded, and the remaining sequences were mapped to their respective Operational Taxonomic Unit (OTU) with the Kraken2 software (v2.1.2) using the pre-indexed Standard database of bacteria, archaea, and viruses. Kraken2 was used to quantify eukaryotic, microbial and viral reads. However, despite human sequence removal, many reads were classified as human by Kraken2. Kraken2 uses k-mers for this classification.^22^ Bracken (v2.6.0) was used to estimate the abundance of the classified OTUs at a genus level.^23^ Statistical analysis of these genera to determine differential expression between UC and controls was done using DESeq2 (v1.4.2).^24^ DESeq2 was also used for normalization of all RNA samples. Genera with row counts less than 32 or a log2 row variance less than 0.2 were removed before DESeq2 analysis. FDR adjusted p-values of less than 0.05 were considered significant. DESeq2 uses an interpretation of FDR/Benjamini-Hochberg, first ranking by p-value, then multiplying each ranked p-value by m/rank.^25^ Omicsbox (v2.2) was used for the generation of the rarefaction curve and the genus diversity curve. The PCoA plot was generated using ggplot in R (v4.1.1). For the rarefaction curves, ggplot2 (v3.4.2) in R (v4.2.2) was used.

## Results

### Patient samples

Mucosal tissue samples were taken at the site of inflammation as described in the Materials & Methods section. Mucosal samples taken from the control subjects showed normal colonoscopy, colon histology, and immunochemistry, with clinical and endoscopic Mayo scores = 0. The age distribution within the groups was 43 ± SD 16 y in the UC group and 52,9 ± SD 16 in the control subjects. The gender distribution within both groups was 22 males and 11 females in the UC group and 10 males and 5 females in the control group.

### Quality control of transcriptional data

The average number of uniquely mapped reads per sequencing run was 85 million reads per sample, representing the entire transcriptome in a mucosal sample. The human depleted transcriptome is available through SRA at BioProject with accession number PRJNA1100419 (https://www.ncbi.nlm.nih.gov/sra/PRJNA1100419). The workflow of this project is outlined in Supplementary Data 1. To ensure that sufficient reads were attained for the microbial composition in mucosal samples to be accurately represented, rarefaction curves were produced for all samples (*n* = 48). Here, the number of expected original taxonomic units (OTUs) was plotted against the number of sequencing reads. The plot shows that the curves all flattened out with an increase in sequencing depth (Supplementary Data 2).

The Shannon and Simpson (Supplementary Data 3) diversity indexes showed that the average species diversity was lower in UC than in controls (Shannon: controls = 0.1, UC = 0.06; Simpson: controls = 0.216, UC = 0.11). A t-test on the Shannon indices for controls versus UC gave a p-value of *p* = 0.0589. The Simpson index t-test gave a p-value *p* = 0.0741. All the findings at the species level are listed in Supplementary Data 4, and at the genus level in Supplementary Data 5.

### Difference in taxonomic composition between UC and controls

**Figure 1.**
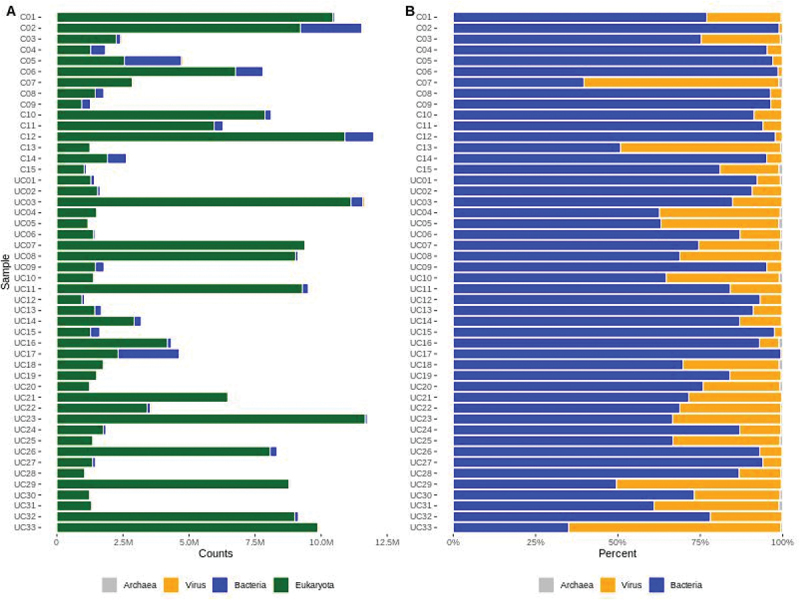
(a) Sequencing read counts for eukaryotes, bacteria, virus, and archaea (x-axis) in all control and UC samples (y-axis). (b) Composition of bacteria, virus and archaea reads in all samples.

An overview of the composition of eukaryotic and prokaryotic (bacteria, virus, and archaea) reads in UC and control samples (C) are presented in Figure 1a. This showed that control samples had on average 656,763 mapped reads versus 202,661 in UC samples. Samples UC17, C2, and C5 had a much higher number of microbial reads than the other samples, with 2,317,748, 2317748, and 2,156,623 reads. The samples UC17 and C5 had a low percentage of eukaryotic reads with 49,9% and 53.5%, while C2 had 79,8% eukaryotic reads (Figure 1a).

**Figure 2.**
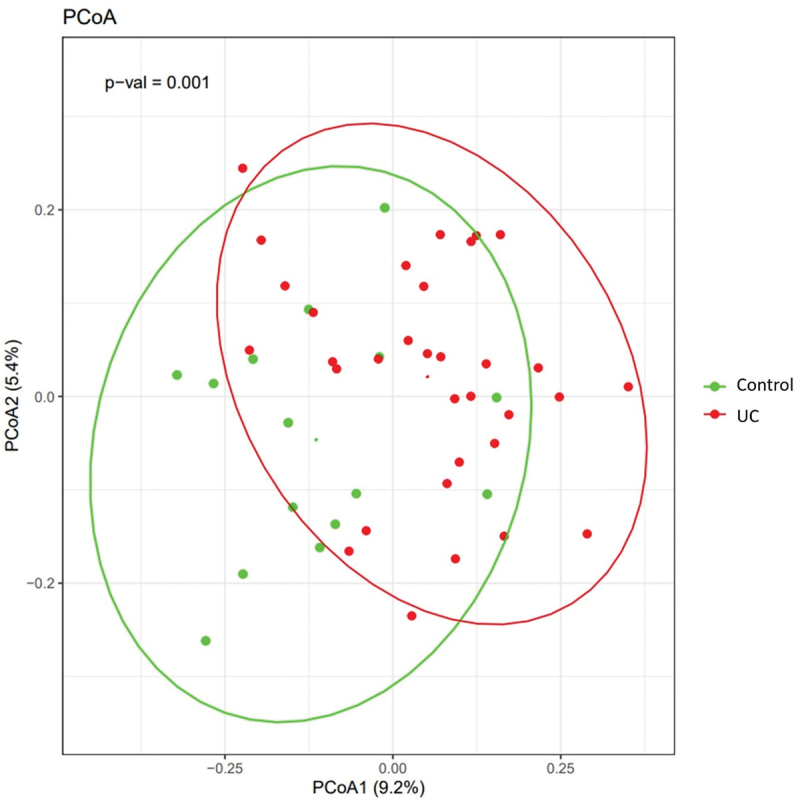
Principal coordinates analysis (PCoA) separating each sample based on bray-Curtis’s dissimilarities between the genus relative expression on a 9.2% PCoA1 ×-axis and a 5.4% PCoA2 y-axis, with UC samples (red) and control samples (green). The ellipses for UC and controls were drawn using a 95% confidence interval. The small red and green dots represent ellipses means.

The percentage of bacteria, viruses, and archaea per sample is shown in Figure 1b. All samples had a low percentage of archaea reads, with six samples (C7, C15, UC5, UC16, UC18 and UC31) having more than 1% of the microbial reads being archaea. The highest percentage (1.15%) of archaea reads were found in sample UC5. Excluding eukaryotic reads, all samples had mostly bacterial reads, while samples C7, UC29, and UC33 had mostly viral reads (59.2%, 50.1% and 64.4 % viral reads). The viral reads ranged from 4846 reads in UC28 to 77,980 reads in UC3, while the percentage of viral reads varied from 0.45% in sample UC17 to 64.4% in sample UC33. There is an overall correlation between the amount of virus and total reads R = 0.48 (p.value = 0.0005). However, this is only true for UC samples, where the correlation between virus and total reads is R = 0.73 (p.value = 1.18e-06). For the controls, the correlation between virus and total reads is *R* = −0.12 (p.value = 0.67). Some samples revealed considerable variations in viral read abundance. For example, the control samples C7 and C13 show high viral proportions despite low total reads (Figure 1). Despite the high proportion of viral reads, samples C7 and C13 are within the 95% confidence interval of the control group as shown in Supplementary Figure S1. To better access viral enrichment relative to the microbial community, the total number of viral reads was divided by the total number of viral and microbial reads. Viral counts are enriched in several UC samples when compared to total viral and microbial counts (Suplementary Figure S2). These results suggest that the correlation between viral reads and total reads is dependent on biological variation. A genus diversity curve was plotted to show how much the microbial diversity of all samples could benefit from additional samples (Supplementary Data 6). Principal coordinate analysis (PCoA) of UC and control samples showed that 9.2% of the dissimilarity was accounted for by PCoA1 on the x-axis and 5.4% by PCoA2 on the y-axis (Figure 2) by using Bray Curtis’s dissimilarities.

### Differences between UC and controls at a genus level

Data analyses revealed 16 differentially expressed genera with a padj <0.05 (Table 1), of which 11 were less transcriptionally active in UC compared to controls, and 5 were higher transcriptionally active in UC compared to controls. The average number of reads (Base mean) is indicative of how common each genus was in individual mucosal samples (Table 1).

**Table 1.** Differential expression of bacterial transcripts based on a genus level for ulcerative colitis (UC) versus controls.

TaxID	Name	^#^Base mean	^¤^log2 FC	^$^lfcSE	^€^Padj
338	*Xanthomonas*	43.7670	2.9820	0.5411	5.00E–06
286	*Pseudomonas*	670.8976	2.2087	0.4893	0.0003
283	*Comamonas*	23.0509	1.5313	0.4701	0.0121
469	*Acinetobacter*	151.8753	1.1112	0.3303	0.0094
106589	*Cupriavidus*	26.5788	0.8900	0.3095	0.0333
423349	*Mucilaginibacter*	32.0820	−1.3478	0.4522	0.0278
1350	*Enterococcus*	68.9868	−1.4292	0.3269	0.0005
2767842	*Lactiplantibacillus*	25.7331	−1.8515	0.5442	0.0085
1016	*Capnocytophaga*	19.0333	−2.0420	0.4440	0.0002
133925	*Olsenella*	32.7144	−2.3434	0.6614	0.0056
1637	*Listeria*	71.2677	−2.5158	0.4271	1.08E–06
1501226	*Romboutsia*	1812.9352	−2.6420	0.7349	0.0051
191303	*Turicibacter*	103.6119	−2.7656	0.8036	0.0077
1485	*Clostridium*	5014.3708	−2.8061	0.5862	0.0002
239934	*Akkermansia*	30.0857	−3.1537	0.7480	0.0006
838	*Prevotella*	1996.3962	−3.4983	0.7570	0.0002

^#^Base mean = average number of reads; ^¤^log2FC = log2 fold change; ^$^lfcSE = log fold change standard error; ^€^padj = p adjusted.

**Figure 3.**
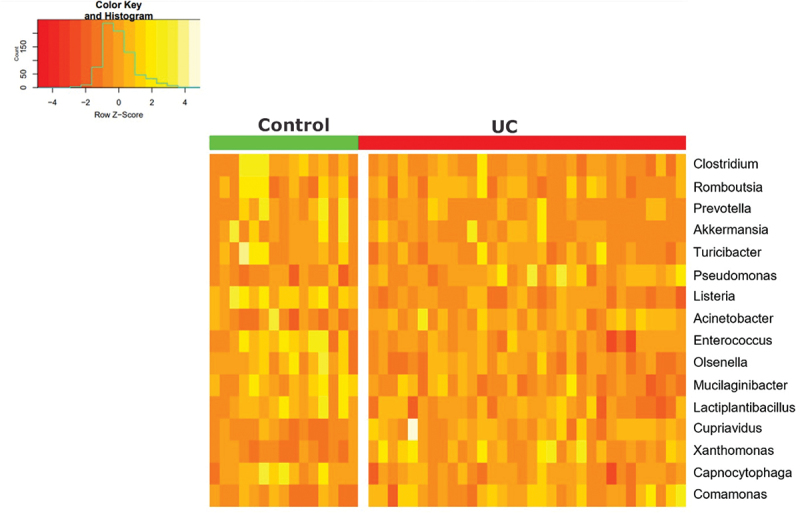
Heatmap visualizing the up- and downregulation of the 16 genera in UC (red) compared to controls (NN) (green). The Z-score represent the number of standard deviations away from the mean, with Z > 0 representing an up-regulated genus in a sample and Z < 0 a down-regulated genus.

**Figure 4.**
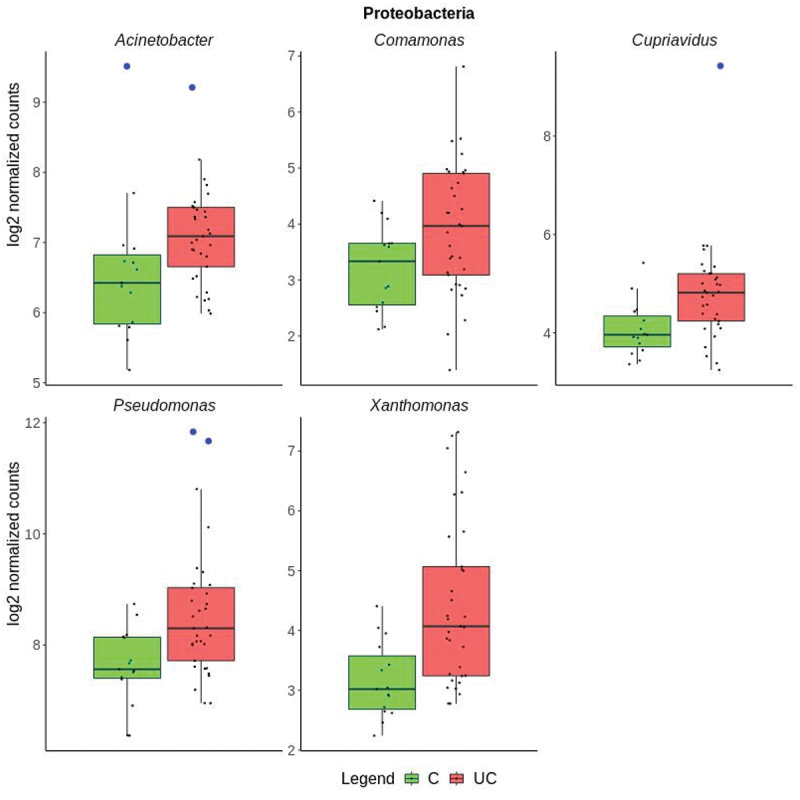
Boxplots of genera in the Proteobacteria phylum with significantly higher expression in ulcerative colitis (UC in red) than controls (C in green), showing the median values and the upper and lower quartiles. Individual samples are marked as black points. The y-axis shows the group log2 expression per genus after normalization. Outliers are indicated by a blue circle.

**Figure 5.**
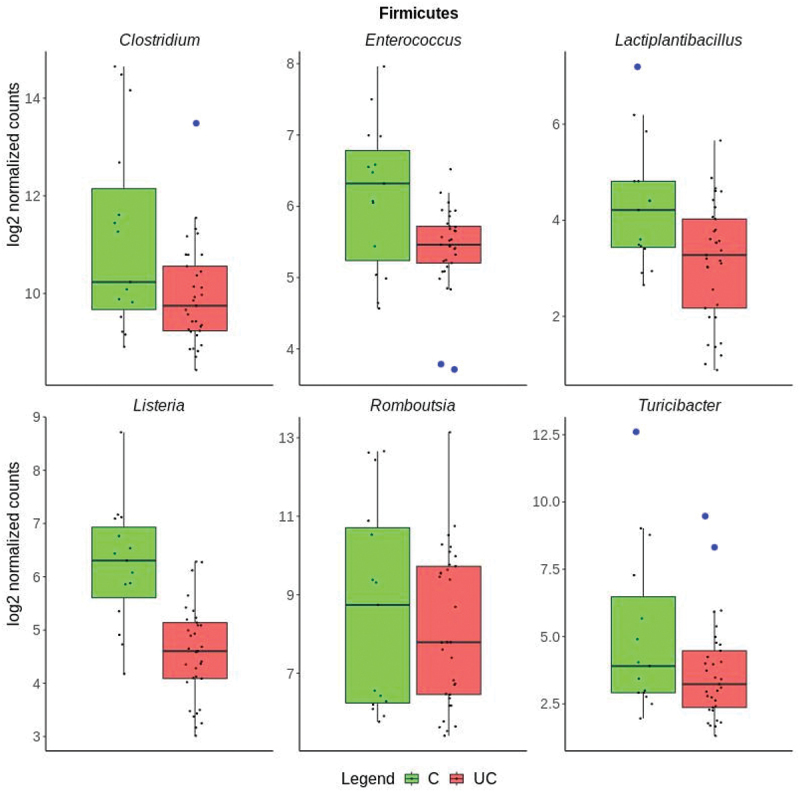
Boxplots of genera in the *Firmicutes* phylum with significantly lower expression in ulcerative colitis (UC in red) than controls (C in green), showing the median values and the upper and lower quartiles. Individual samples are marked as black points. The y-axis shows the group log2 expression per genus after normalization. Outliers are indicated by a blue circle.

**Figure 6.**
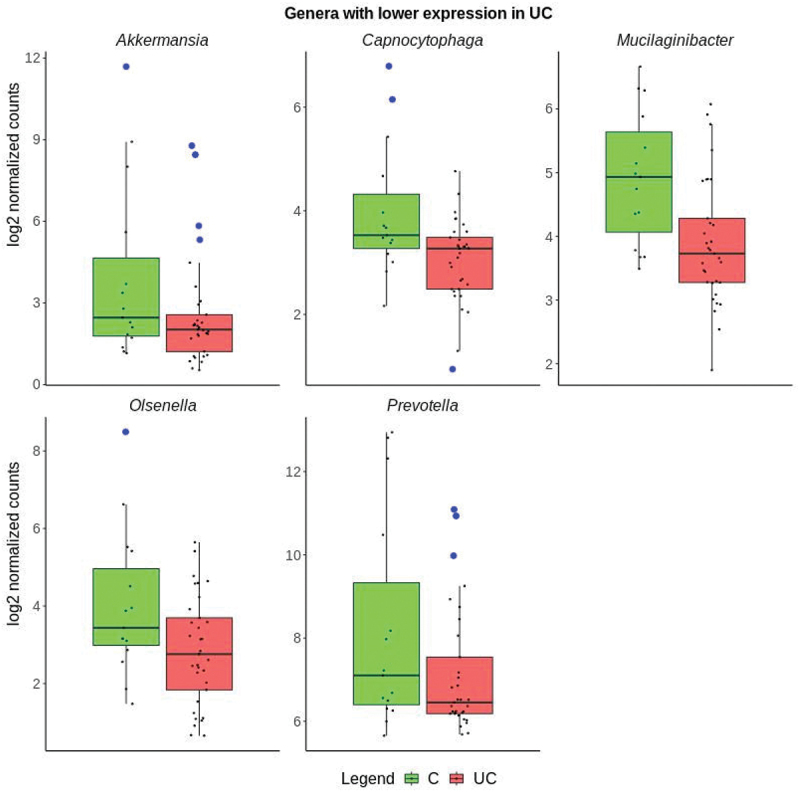
Boxplots of genera in phyla that with significantly lower expression in ulcerative colitis (UC in red) than controls (C in green), showing the median values and the upper and lower quartiles. Individual samples are marked as black points. The y-axis shows the group log2 expression per genus after normalization. Outliers are indicated by a blue circle.

The differences in transcriptional activity of 16 different genera for 33 UC and 15 control samples is visualized in Figure 3. Five genera of the Proteobacteria phylum were more expressed in UC compared to controls including *Pseudomonas, Acinetobacter, Cupriavidus, Xanthomonas*, and *Comamonas* (Figure 4). The largest differences between UC and controls were observed for *Acinetobacter* and *Cupriavidus*. Six of the genera that were less transcriptionally active in UC than controls were in the *Firmicutes* phylum including *Listeria, Clostridium, Turicibacter, Lactiplantibacillus, Roumboutsia*, and *Enterococcus*. (Figure 5). The remaining five genera, *Prevotella, Mucilaginibacter*, *Capnocytophaga*, belonging to the *Bacteroidetes* phylum, *Akkermansia*, belonging to the Verrucomicrobia phylum, and *Olsenella* belonging to the Actinobacteria phylum were less transcriptionally active in UC (Figure 6). Human Cytomegalovirus (*human betaherpesvirus 5*) was only found in four UC patient samples (Supplementary Data 4).

## Discussion

The nature of the chronic inflammatory disease ulcerative colitis (UC) is believed to be a result of gut microbiota alterations among many other factors.^26^ UC has been associated with a shift in the balance between commensal and potentially pathogenic microorganisms followed by decreased microbial diversity.^27–29^ In this study, a meta-transcriptomic approach was applied to determine the actively transcribing microbial community present at the active site of inflammation in mucosal biopsies of patients with active UC. The objective of the study was to identify microbial composition from actively transcribed sequences (RNA) rather than using a genomic shotgun approach (DNA). To help enrich microbial detection, human ribosomal RNA was depleted during the library preparation process, which makes up 80–90% of the total RNA.^30^

The obtained results are believed to be useful for mapping the active functional profile of microbial communities and are further discussed below. The commonly used 16S rRNA gene sequencing approach provides no information on transcriptional activity outside of the 16S rRNA gene, and less abundant but biologically meaningful bacterial species might remain undetected.^31,32^ Some species have been found to be more transcriptionally active than reflected by their genomic abundance or *vice versa*.^13^ The rarefaction curves indicated a good estimation of diversity at a genus level. At the species level, there are on average far fewer reads and greater variation between samples. Therefore, given the small cohort, we are much more confident in reporting results at a genus level than species level.

The meta-transcriptomic approach used here revealed an increased transcriptional activity of *Proteobacteria* (Figure 4) which contrasts with results obtained by 16S rRNA gene sequencing of mucosal biopsies.^33^ *Proteobacteria* have been identified as bacteria that prompt endotoxemia and metabolic disorders in human GI tracts.^34^ It is hereby noted that the transcriptional activity of Proteobacteria is not always accompanied by their abundance.^33^

16S rRNA gene and shotgun sequencing of fecal samples have been widely used to determine the microbiota composition in inflamed colonic tissue.^35^ However, fecal samples represent the entire luminal microbiota and might not include microorganisms that have translocated across the epithelial barrier.^36^ Both methods do not represent the transcribing and actively functioning bacterial genome at the site of inflammation. Therefore, it is important to note that colonic mucosal tissues were taken directly at the site of inflammation and obtained from uncompromised treatment-naïve UC patients. In addition, 16S rRNA gene sequencing reveals only a part of bacterial genomes and results in incomplete species determination.^37^ For example, the observed decrease in the expression of *Olsenella* (Figure 6, Table 1) contrasts with findings from a study using 16S rRNA gene sequencing of fecal samples.^38^ The species in the *Olsenella* genus has recently been isolated, its significance in UC remains unclear.^39^

An initial principal coordinate analysis (PCoA) revealed differences in the microbial compositions of UC and control samples (Figure 2) with sufficient coverage at the genus level (Supplementary Data 2 & 6). Significant differences in expression at the genus level between UC and control are depicted in Table 1. Mucosal tissues are heterogenous and composed of many different cell types which might account for the observed varying microbial and human reads in some of the samples (Figure 1). Consistent with other studies, a decrease in microbial diversity was found in active UC compared to controls (Figure 1, Supplementary Data 3) which indicates a less functional diversity of the microbiome.^40,41^

Two genera containing opportunistic pathogens, *Acinetobacter* and *Comamonas*, were found to be significantly increased in UC compared to controls (Figure 4, Table 1). Studies using 16S rRNA gene sequencing of colonic biopsies and stool samples indicate that an increase of abundance of *Acinetobacter* is associated with inflammation^42,43^ It is hereby noted that an increase of *Acinetobacter* in distal colon mucosal swabs compared to fecal samples has been recently reported.^44^ The *Acinetobacter* genus includes *A. baumannii*, which is prevalent in hospital acquired infections^45^ (Supplementary Data 4). Additionally, a role for *Comamonas* in UC inflammation can be implied, as indicated by increased expression in active UC compared to controls, which has not been previously reported (Figure 4, Table 1). As an environmental pathogen, *Comamonas* species has a high degree of genetic flexibility, and the ability to survive in ecological niches, making them a dangerous candidate for mild but persistent infections, especially among individuals with predisposing conditions.^46^ In this context, recent clinical reports indicate a potential role for *Comamonas* in gastroenteritis, intra-abdominal infections, and bacteriemia.^47–50^

Several genera containing commensal bacteria were found to be differentially expressed in mucosal UC samples compared to controls (Table 1). Among these were *Akkermansia*, represented by *A. muciniphila* at the species level (Supplementary Data 4). *A. muciniphila* regulates gut barrier function by degrading mucin and producing short chain fatty acids (SCFA) used as substrate by other commensal bacteria.^51–53^ A lower abundance of *A. muciniphila* has been shown to be accompanied by a decrease of mucosal barrier function during inflammation.^52^ The here observed decreased levels of *A. muciniphila* (Figure 6) are consistent with results observed in fecal samples.^54,55^

A depletion of the transcriptional activity of *Prevotella* at a genus level was observed in active UC (Figure 6) and has been previously reported.^56^ *Prevotella* are normally considered commensal bacteria. However, several species of *Prevotella* can worsen inflammation.^57^ For example, *P. intestinalis* has a negative effect on the gut by reducing the amount of short chain fatty acids (SCFAs), and thereby the production of IL-18,^58^ which is important to control the barrier function in UC.^59^ Mice colonized with *P. copri* showed an increased susceptibility to chemically induced colitis.^60^ The observed decrease in both the abundance and transcriptional activity of this genus might indicate that *Prevotella* is not among the bacteria that exacerbate inflammation. However, a causation or correlation of the abundance and transcriptional activity of *Prevotella* in active UC is still debatable.

The gut microbiota also contains viruses that might also play a part in UC pathogenesis.^61^ Here, human cytomegalovirus (CMV) (*Human betaherpesvirus 5*) was only found in four UC samples (Supplementary Data 4). Significantly higher DNA loads of CMV have been detected in diseased compared to non-diseased mucosa of IBD patients which supports our findings.^62^ CMV has been implicated in flare-ups of UC that are resistant to treatment.^63^ In general, the observed variations in viral abundance suggest biological virome alterations found in the healthy gut virome^64^ and in ulcerative colitis^65–67^ (Suplementary Figure S2).

A limitation of this work is eliminating of all human genetic material from whole transcriptomes, which is a challenging task with the current available *in silico* methods. Most of the sequenced samples consist of about 95% human genetic material. Depletion of human ribosomal RNA is the first step in the sequencing library preparation to reduce a part of human reads. However, even after rRNA depletion, the vastly more abundant host mRNAs and non-coding RNAs dwarf the bacterial mRNAs.^68^ Removing the remaining host RNA may have an effect of removing a small but considerable proportion of microbial RNA. It has been shown that bacterial miRNAs may target the human transcriptome.^69^ Potentially, these bacterial miRNAs could be depleted by host RNA removal. The effects of read depth can be seen at the species level, where there is considerable variation between samples. This may be a result of low read depth caused by an oversaturation of human reads or may represent the microbial diversity between samples. Therefore, rarefaction curves were generated (Supplementary Data 2) to ensure sufficient sequencing depth for genus-level analysis regardless of this limitation. The intention of using mucosal colonic biopsies was to study the actively transcribed microbial RNA present at the site of inflammation, thereby defining the microbial population that has potentially penetrated the gut barrier. The microbial profiles observed likely reflect mucosa-associated and epithelial-adherent bacteria rather than the broader luminal community and thus may highlight taxa more directly involved in host–microbe interactions at the site of inflammation.

It is difficult to determine if the changes in microbial expression are a cause or an effect on UC inflammation.^2^ It is quite difficult to determine whether an individual’s microbiome is an outlier with respect to healthy individuals, as microbiome profiles vary widely. This is especially true in a complex disease such as ulcerative colitis, as the microbiome of UC patients may be indicative of biologically relevant outliers when compared to healthy individuals. For individual genera comparisons, outliers are indicated (Figures 4–6). In addition, most bacteria lack fully annotated transcriptomes and might be missing non-coding RNA and untranslated regions.^12^ The tissue heterogeneity of mucosal biopsies and the small sample size of the cohort might explain why some samples in this study had a higher microbial content than others (Figure 1).

## Conclusion

The meta-transcriptomics approach demonstrated herein indicates that extracting the active microbial composition from mucosal biopsies is indeed feasible. Identifying environmental pathogens acquired by the host, specifically *Comamonas*, may yield new insights into the pathogenesis of ulcerative colitis (UC). These findings potentially pave the way for future targeted studies examining host–microbe interactions in individual patients with UC.

## Supplementary Material

Supplementary Figure 2.pdf

Supplementary Data 6.docx

Supplementary Data 3.docx

Supplementary Data 4.xlsx

Supplementary Figure 1.pdf

Supplementary Data 5.xlsx

Supplementary Data 1.docx

Supplementary Data 2.docx

## Data Availability

The human depleted transcriptome is available through the NCBI Sequence Read Archive (SRA) at BioProject accession number PRJNA1100419, at https://www.ncbi.nlm.nih.gov/sra/PRJNA1100419.
