# Supplementary material for: Actively expressed microbiota in mucosal biopsies of treatment-naïve ulcerative colitis patients
Source: Gut Microbes Rep. 2025 Jun 5;2(1):2512763. doi: 10.1080/29933935.2025.2512763 (PMC12940147; doi:10.1080/29933935.2025.2512763)
Supplement: Supplementary Figure 2.pdf [file KGMR_A_2512763_SM6886.pdf]

Supplementary Figure 2

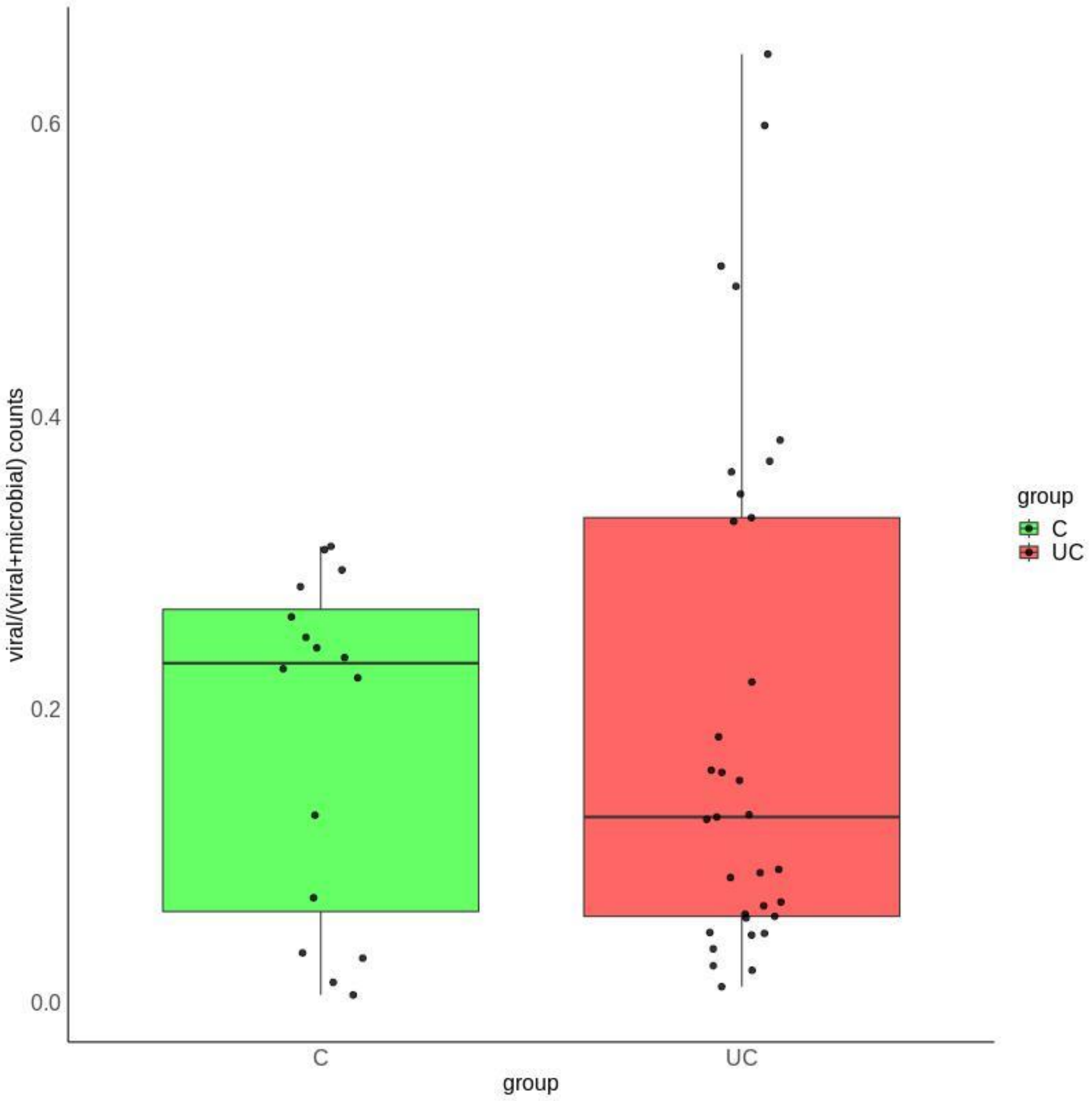

Supplementary figure 2 shows a ratio of total viral counts divided by total viral and total microbial counts. Individual samples are indicated in black. The green boxplot shows the control samples (C), the red boxplot shows the ulcerative colitis samples (UC).
