# Supplementary material for: Actively expressed microbiota in mucosal biopsies of treatment-naïve ulcerative colitis patients
Source: Gut Microbes Rep. 2025 Jun 5;2(1):2512763. doi: 10.1080/29933935.2025.2512763 (PMC12940147; doi:10.1080/29933935.2025.2512763)
Supplement: Supplementary Data 6.docx [file KGMR_A_2512763_SM6885.docx]

**Supplementary Data 6**

**Diversity Curve**

Figure text: Diversity curve showing the cumulative number of OTUs (y-axis) as a function of the number of samples (x-axis). correlation between the number of genera (y-axis) and the number of samples (x-axis) with a 95% CI. A flattening curve means the number of genera remains the same with additional samples.

**
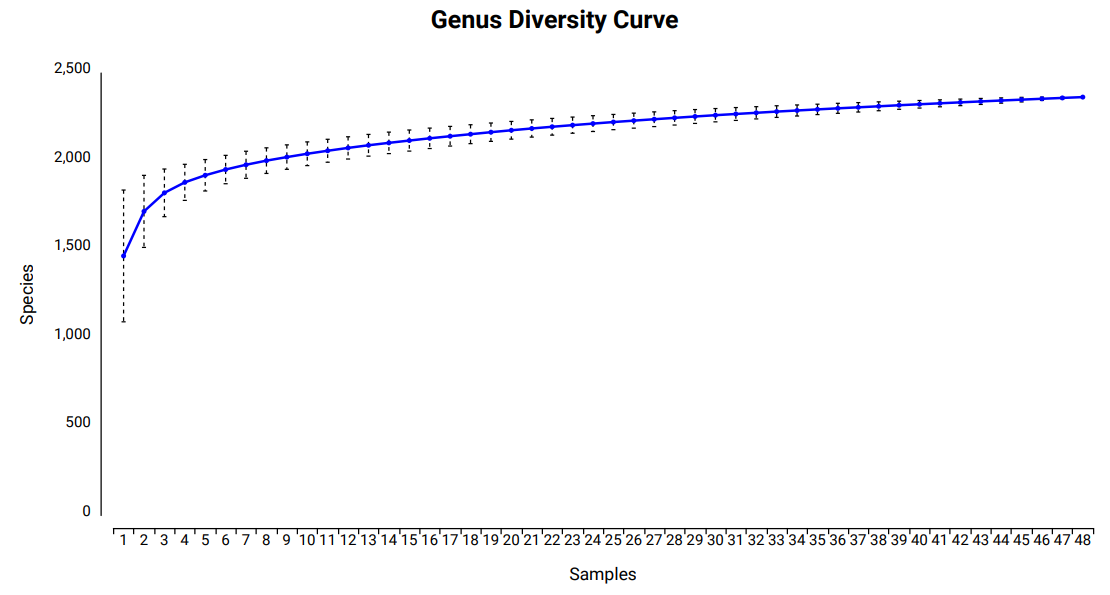
**
