# Supplementary material for: Actively expressed microbiota in mucosal biopsies of treatment-naïve ulcerative colitis patients
Source: Gut Microbes Rep. 2025 Jun 5;2(1):2512763. doi: 10.1080/29933935.2025.2512763 (PMC12940147; doi:10.1080/29933935.2025.2512763)
Supplement: Supplementary Data 3.docx [file KGMR_A_2512763_SM6884.docx]

**Supplementary Data 3**

**Shannon index and Simpson index** Table with Simpson and Shannon index as a measure of diversity (y-axis) for all control (C) and UC samples, with t-tests.

| Sample | Simpson index | Shannon index |
| --- | --- | --- |
| C1 | 0,0294 | 0,0154 |
| C2 | 0,0278 | 0,0137 |
| C3 | 0,2236 | 0,0984 |
| C4 | 0,4386 | 0,1994 |
| C5 | 0,5327 | 0,2401 |
| C6 | 0,2032 | 0,0876 |
| C7 | 0,0979 | 0,0436 |
| C8 | 0,2412 | 0,1007 |
| C9 | 0,364 | 0,1799 |
| C10 | 0,064 | 0,032 |
| C11 | 0,0885 | 0,0456 |
| C12 | 0,1464 | 0,0676 |
| C13 | 0,0922 | 0,0427 |
| C14 | 0,4176 | 0,1849 |
| C15 | 0,2753 | 0,124 |
| UC1 | 0,2467 | 0,1177 |
| UC2 | 0,1018 | 0,0519 |
| UC3 | 0,0821 | 0,0388 |
| UC4 | 0,1185 | 0,0545 |
| UC5 | 0,0794 | 0,04 |
| UC6 | 0,1138 | 0,0564 |
| UC7 | 0,0265 | 0,0134 |
| UC8 | 0,0369 | 0,0189 |
| UC9 | 0,3026 | 0,1382 |
| UC10 | 0,0767 | 0,0384 |
| UC11 | 0,0537 | 0,0278 |
| UC12 | 0,191 | 0,0952 |
| UC13 | 0,2407 | 0,1063 |
| UC14 | 0,1742 | 0,0804 |
| UC15 | 0,2743 | 0,1213 |
| UC16 | 0,1696 | 0,0717 |
| UC17 | 0,82 | 0,3288 |
| UC18 | 0,1256 | 0,0561 |
| UC19 | 0,0672 | 0,0351 |
| UC20 | 0,1423 | 0,0646 |
| UC21 | 0,0268 | 0,0147 |
| UC22 | 0,1075 | 0,0515 |
| UC23 | 0,0359 | 0,0175 |
| UC24 | 0,1547 | 0,0743 |
| UC25 | 0,0606 | 0,0313 |
| UC26 | 0,0701 | 0,0318 |
| UC27 | 0,1199 | 0,0564 |
| UC28 | 0,068 | 0,0361 |
| UC29 | 0,0357 | 0,0172 |
| UC30 | 0,0569 | 0,0303 |
| UC31 | 0,0771 | 0,0383 |
| UC32 | 0,0398 | 0,0213 |
| UC33 | 0,0316 | 0,015 |
| t-test | Simpson t test | Shannon t test |
| t-test | 0,07411679 | 0,05890855 |
