# Supplementary material for: Actively expressed microbiota in mucosal biopsies of treatment-naïve ulcerative colitis patients
Source: Gut Microbes Rep. 2025 Jun 5;2(1):2512763. doi: 10.1080/29933935.2025.2512763 (PMC12940147; doi:10.1080/29933935.2025.2512763)
Supplement: Supplementary Figure 1.pdf [file KGMR_A_2512763_SM6882.pdf]

Supplementary Figure 1

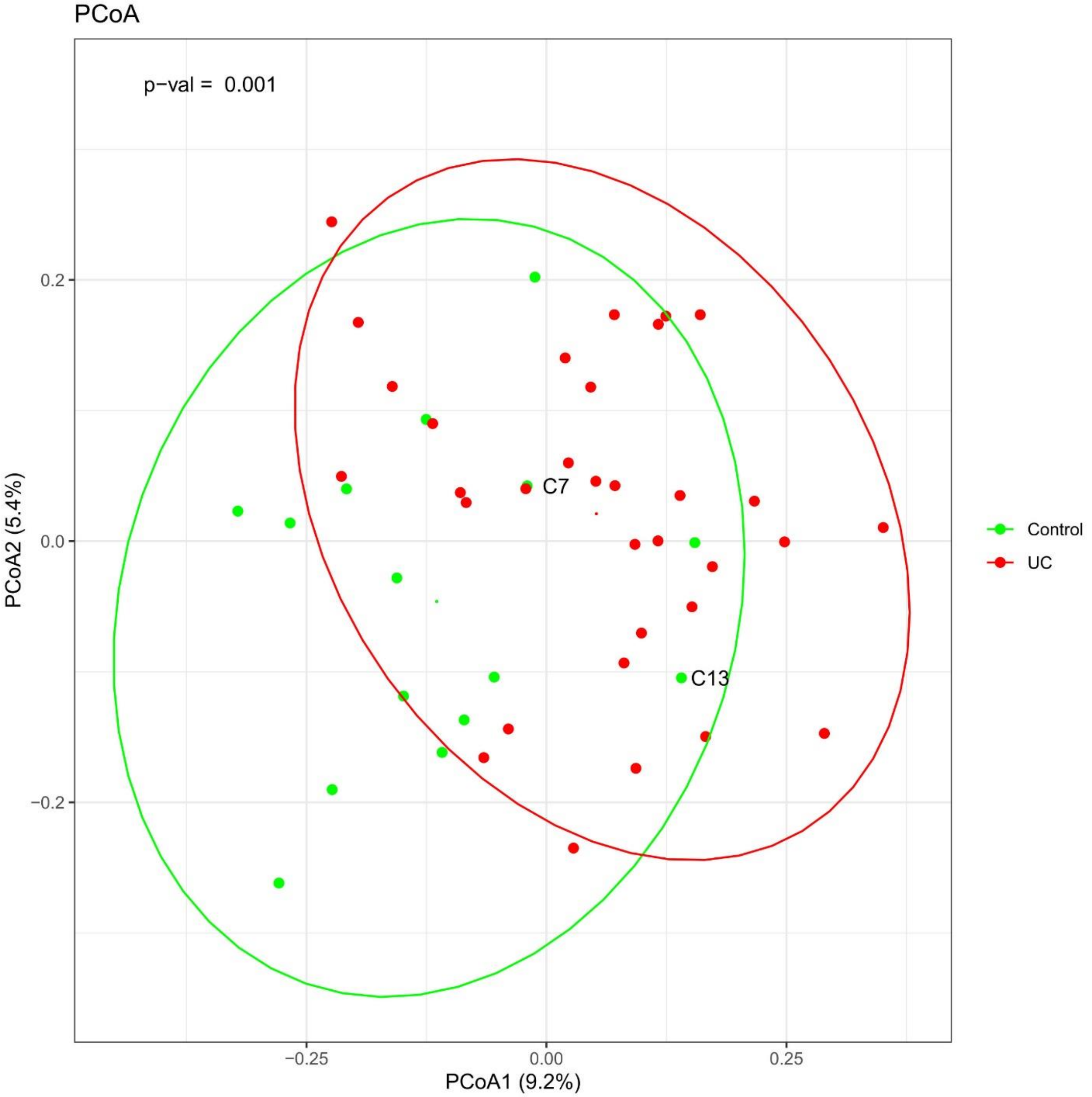

Principal coordinates analysis (PCoA) separating each sample based on Bray-Curtis's dissimilarities between the genus relative expression on a 9.2% PCoA1 x-axis and a 5.4% PCoA2 y-axis, with UC samples (red) and control samples (green). The ellipses for UC and controls were drawn using a 95% confidence interval. The small red and green dots represent ellipses means. Samples with lower read counts where viral reads constitute a higher proportion are indicated on the plot (C7 and C13).
