# Supplementary material for: Actively expressed microbiota in mucosal biopsies of treatment-naïve ulcerative colitis patients
Source: Gut Microbes Rep. 2025 Jun 5;2(1):2512763. doi: 10.1080/29933935.2025.2512763 (PMC12940147; doi:10.1080/29933935.2025.2512763)
Supplement: Supplementary Data 1.docx [file KGMR_A_2512763_SM6880.docx]

**Supplementary Data 1**

**Flowchart**

Flowchart of meta-transcriptomic analysis, from sample retrieval through data processing.

**
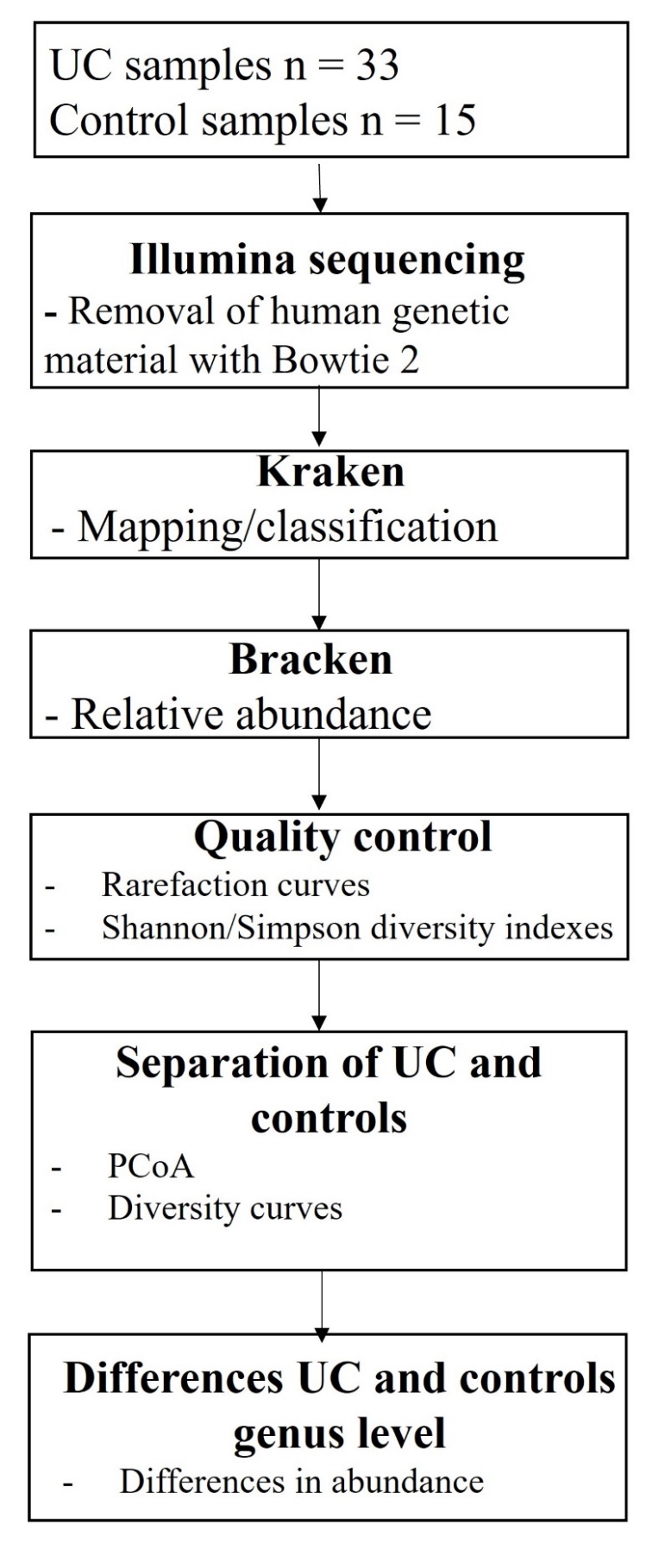
**
