# Supplementary material for: Actively expressed microbiota in mucosal biopsies of treatment-naïve ulcerative colitis patients
Source: Gut Microbes Rep. 2025 Jun 5;2(1):2512763. doi: 10.1080/29933935.2025.2512763 (PMC12940147; doi:10.1080/29933935.2025.2512763)

**Supplementary Data 2**

**Rarefaction Curves**

Rarefaction curves of all samples in the study at a genus level. Each curve represents the number of expected OTUs (y-axis) plotted against the number of reads attained through Illumina sequencing (x-axis). Each curve represents one sample. A horizontal asymptotic curve indicates that a good estimation of diversity has been obtained.


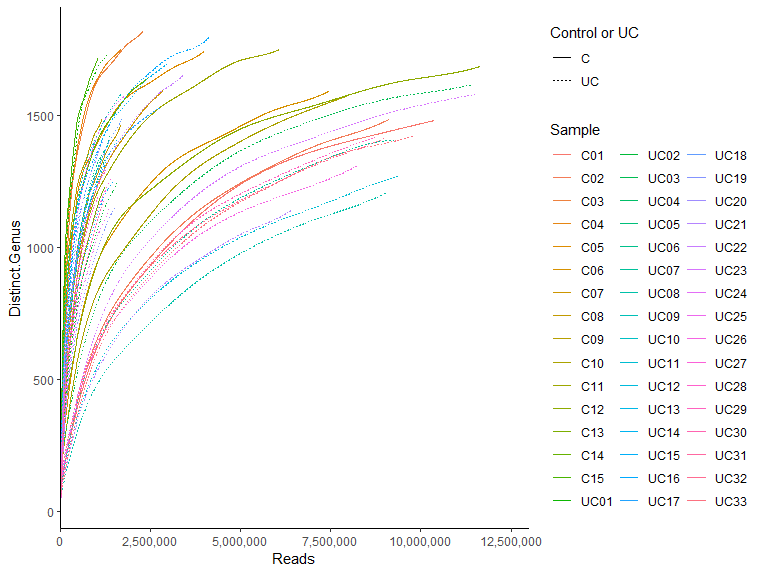

Supplement: Supplementary Data 2.docx [file KGMR_A_2512763_SM6879.docx]
